# A framework for assessing clinical trial site readiness

**DOI:** 10.1017/cts.2023.541

**Published:** 2023-05-08

**Authors:** John B. Buse, Christopher P. Austin, S. Claiborne Johnston, Freda Lewis-Hall, Andrew N. March, Carolyn K. Shore, Pamela Tenaerts, Joni L. Rutter

**Affiliations:** 1 Department of Medicine, University of North Carolina School of Medicine, Chapel Hill, North Carolina, USA; 2 Flagship Pioneering, Cambridge, Massachusetts, USA; 3 Harbor Health, Austin, TX, USA; 4 Retired from Pfizer Inc., USA; 5 National Academies of Sciences, Engineering, and Medicine, Washington, District of Columbia, USA; 6 Medable, Inc, USA; 7 National Center for Advancing Translational Sciences, National Institutes of Health, Bethesda, Maryland, USA

**Keywords:** Clinical trial sites, site readiness practices, quality improvement, evaluation, adoption and implementation

## Abstract

Clinical trial processes are unnecessarily inefficient and costly, slowing the translation of medical discoveries into treatments for people living with disease. To reduce redundancies and inefficiencies, a group of clinical trial experts developed a framework for clinical trial site readiness based on existing trial site qualifications from sponsors. The site readiness practices are encompassed within six domains: research team, infrastructure, study management, data collection and management, quality oversight, and ethics and safety. Implementation of this framework for clinical trial sites would reduce inefficiencies in trial conduct and help prepare new sites to enter the clinical trials enterprise, with the potential to improve the reach of clinical trials to underserved communities. Moreover, the framework holds benefits for trial sponsors, contract research organizations, trade associations, trial participants, and the public. For novice sites considering future trials, we provide a framework for site preparation and the engagement of stakeholders. For experienced sites, the framework can be used to assess current practices and inform and engage sponsors, staff, and participants. Details in the supplementary materials provide easy access to key regulatory documents and resources. Invited perspective articles provide greater depth from a systems, DEIA (diversity, equity, inclusion, and accessibility) and decentralized trials perspective.

## Introduction

The COVID-19 pandemic laid bare the inefficiencies and health inequities of the US clinical trials enterprise, which impede delivery of promising new therapies to the public. Despite the unprecedented success of Operation Warp Speed and the Accelerating COVID-19 Therapeutic Interventions and Vaccines (ACTIV) trials, early in the pandemic, insufficient trial infrastructure and a lack of coordination hampered the ability of the US clinical trials enterprise to adequately respond quickly. Going forward, there is a need to ensure clinical trial site readiness to meet timelines and recruitment goals, and to speed definitive and actionable evidence relevant to clinical care. Despite ongoing efforts [1,2], the clinical trials enterprise has not yet adequately expanded participation beyond academic research centers and professional research sites. This manuscript is meant to facilitate the engagement of new sites, provide a framework for health systems to expand their clinical trials footprint, and serve as a resource for engagement of new staff, sponsors, and participants.

Trial access remains a challenge for frontline clinicians and people living with disease [3]. One problem is that the process for initiating a clinical trial site involves multiple complex qualifications [4]. Sponsors tend to use the same sites repeatedly [5] – a practice that offers operational and strategic advantages at the expense of generalizability and the inclusion of underserved communities. Local, regional, national, and international regulations add additional hurdles when it comes to streamlining, sunsetting, or harmonizing site qualifications across regions and studies.

While compliance with good clinical practice (GCP) helps ensure the ethical and scientific quality of clinical trials, sites are also required to comply with sponsor-specific qualifications as detailed in feasibility questionnaires, which creates inefficiencies for sites, sponsors, researchers, and the public [6]. In the absence of consistent and transparent application of baseline qualifications for clinical trial site readiness, the process of site initiation remains fragmented and duplicative. Potential trial sites are burdened by having to repeatedly define quality and determine how to best demonstrate site readiness and meet expectations across different sponsors and studies. This creates particular barriers to participation for community-based sites, which may require additional staff support, infrastructure, and processes to directly and effectively engage in the clinical trials enterprise. The proposed framework provides a harmonized template for sponsors and sites to enhance diversity in recruitment through expansion of ready clinical trial sites.

Several organizations, including the Society for Clinical Research Sites’ Site Qualification Training initiative [7] and the Site Accreditation and Standards Institute [8], provide guidance and resources for clinical trial sites to improve the quality and efficiency of trials. Though focused on oncology clinical trials, the American Society of Clinical Oncology has also developed a set of exemplary attributes for trial sites [9].

The establishment and adoption of a core set of site readiness practices that are applicable across trial sites, irrespective of size, geography, or clinical specialty, should help streamline the process of site assessment for organizations, research teams, sponsors, and participants. A core set of site readiness practices clarifies requirements, reduces duplicative efforts, streamlines feasibility assessments and qualification processes, and diversifies the types of sites that may engage in clinical trials and the populations the trials serve.

For the purposes of this article, a clinical trial site is considered a traditional brick and mortar site: it is the physical location(s) of a clinical facility (e.g., academic medical center, clinic, hospital) where a principal investigator (PI), and those working under the PI’s direction, conduct in-person study activities with trial participants. A site/clinical organization (e.g., hospital) can consist of many research sites (e.g., cardiology and oncology divisions), including community-based practices associated with and/or owned by the organization. However, the site would not include locations where participants complete digital assessments (e.g., electronic patient-reported outcomes), or undergo routine clinical assessments that could be included in trial databases (e.g., labs, procedures, or inpatient/outpatient medical encounters), such as local labs or local health care provider facilities. A forthcoming perspective will focus on decentralized trials (Tenaerts P, Hernandez AF, Lipset C. Clinical trial site readiness for decentralized trials – Fitting trials into today’s world. *J Clin Transl Sci.*, in review).

The site readiness practices laid out in this article are a simplified and refined set based on existing qualifications required by sponsors which, taken together, are intended to reflect the minimum requirements necessary for a traditional site to effectively conduct definitive trials in accordance with GCP and similar site qualification requirements used by sponsors. Additional resources are provided in Supplement A, which provides greater depth and access to specific training materials. These site readiness practices are intended to be acceptable to any sponsor and broadly applicable to sites regardless of the organization or research team. It is important to note that the site readiness practices are intentionally elementary as they do not take into consideration study-specific protocols or sponsor-specific qualifications that may apply to a particular clinical trial. However, they are designed to be flexible and modifiable to accommodate emerging clinical trial models, including master protocols, platform-based trial designs, or decentralized clinical trials. This article was developed by the Clinical Trial Site Standards Harmonization action collaborative, an ad hoc activity associated with the Forum on Drug Discovery, Development, and Translation at the National Academies of Sciences, Engineering, and Medicine. This article does not necessarily represent the views of any one organization, the Forum, or the National Academies and has not been subjected to the review procedures of, nor is it a consensus study report or product of the National Academies.

## Methods

Fifty documents used for assessing, monitoring, and auditing US-based clinical trial sites were collected by MMS Holdings,^a^ a global clinical research organization, from 14 research sponsors of clinical trials, including eight pharmaceutical companies, three contract research organizations (CROs), two government agencies, and one academic institution. From these documents, 217 trial site qualifications were extracted covering initial site assessment, site monitoring, site audit, and site closeout checklists. An analysis was conducted by MMS Holdings to identify similarities, differences, and gaps across the 217 trial site qualifications and compare to the International Council for Harmonisation Good Clinical Practice (ICH GCP) guidelines [10]. Site qualifications were categorized based on an initial set of seven domains: site management, infrastructure, continuous quality improvement, human research protection, study management, data management, and study team/investigator.

A small group^b^ consisting of clinical trial experts, including representatives from government, academic health and research systems, pharmaceutical companies, and CROs, were asked to review the catalog of 217 clinical trial site qualifications to identify qualifications that would indicate a clinical trial site’s readiness to support a standard clinical trial from start to finish.

A modified Delphi method was used to select the site qualifications that would be necessary to support clinical trial site readiness. To conduct the review, members of the small expert group completed a series of three online questionnaires, each focused on two to three domains. In each questionnaire, participants indicated the degree to which they considered each site qualification to be important for demonstrating a clinical trial site’s basic readiness to conduct a clinical trial from beginning to end on a scale of 1 (not at all important) to 5 (absolutely essential). Participants also identified additional site qualifications that could be included within each domain. Throughout this process, participants were encouraged to consult with colleagues to solicit additional input. Following each questionnaire, the data were compiled and distributed to the small group members for review and consideration. Over 2017 and 2018, clinical trial experts^c^ then participated in discussions to review the results and further refine the set of site qualifications and domains and consider common principles. The site qualifications identified through this process are the basis for the site readiness practices discussed in this article.

**Figure 1.**
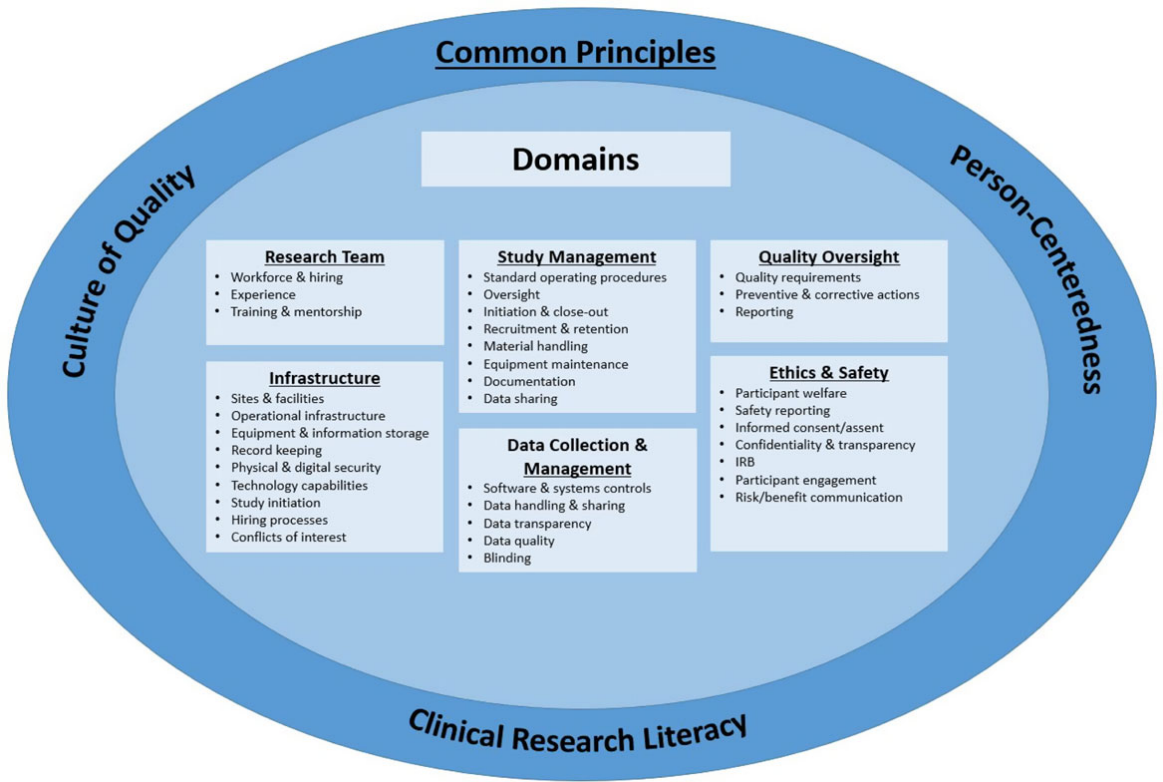
Common principles and domains for site readiness practices for clinical trial site readiness.

The language for these site readiness practices was refined, and a framework developed across six domains: research team, infrastructure, study management, data collection and management, quality oversight, and ethics and safety (Fig. 1). Finally, a subset of the working group participants^d^ carried out a final round of review of the site readiness practices, domain definition, terminology, and common principles.

## Foundational Common Principles

Three principles – a culture of quality, clinical research literacy, and person-centeredness – cut across all of the site readiness practices and are foundational for sites as they ready themselves to conduct clinical trials. In-depth review of these areas is beyond our scope, but brief descriptions of each principle is provided, along with references to recent work that provide additional detail on how they might be applied in practice.

### Culture of Quality

A site possesses a culture of quality when it establishes and demonstrates adherence to processes designed for error prevention or “getting it right the first time” and a process of continuous and incremental improvements. A starting point is setting standards of quality and establishing supporting systems and processes to evaluate and manage quality goals. Sites must have the ability to measure any errors that occur, identify root causes, and apply process improvements for continuous improvement [11–13].

### Clinical Research Literacy

Clinical research literacy – an understanding and appreciation for the value, importance, and processes of clinical research at all levels of an organization – enables informed engagement among stakeholders (the public, potential site PIs and research team members, and health care system leaders) within the clinical research enterprise. For the public, special attention should be paid to increase the understanding of the concepts and terms such as “clinical research” and “clinical trials” as well as where studies take place, how long it takes to develop and test a new treatment, and safeguards for study participants. For PIs and the research team, focus on recognition of the elements that make up a valid research endeavor, requirements for regulatory-grade trials, and the differences in approach for clinical practice versus clinical trials is required across the trial workforce and the clinical enterprise. Further resources in clinical research literacy are available [14].

### Person-Centeredness

Inherent in conducting ethical trials is respect for the rights and welfare of trial participants. It is imperative that clinical trials address the needs and concerns of the general public and maintain flexibility to ensure the well-being of trial participants. Researchers should regard participants as equal partners in research. Engagement with trial participants, communities, and the stakeholders that represent their interests should start early in the process, before initiating a clinical trial and ideally when determining research questions. Participant, community, and stakeholder engagement must be sustained, continuing through the dissemination of study results and between individual clinical trials. Trials that are person-centered also acknowledge health disparities and consciously promote health equity through recruiting diverse trial participants and staff, who reflect the demographics of the disease burden. Detailed treatment of the topic is beyond the scope of this publication and an evolving area of scholarship [15,16].

## Site Readiness Practices for Clinical Trial Sites

The 40 site readiness practices described in this document are organized and consolidated into 6 domains: Research Team, Infrastructure, Study Management, Data Collection & Management, Quality Oversight, and Ethics & Safety. The article proceeds to briefly describe each domain, followed by a listing of the site readiness practices, which are compiled in Supplement B.

The site readiness practices in this article are not new concepts to clinical trial sites, but variability in sites’ adoption of the clinical trial practices as well as potential future advances in clinical trial practices may warrant a renewed examination of the framework. There is a wealth of guidance materials and resources to assist sites in adopting this framework to aide in self-assessment. Supplement A provides clinical trial sites with links to resources to guide the implementation of the site readiness practices from various trusted organizations and agencies. Moreover, Supplement A offers a non-exhaustive list of ways to demonstrate and document compliance with each of the site readiness practices, which sites can subsequently use to demonstrate the quality of their program to study sponsors, regulators, workforce recruits, participants, and the public.

### Research Team

A clinical site research team may include a principal/sub-investigator(s), clinical research associates, research nurses, data managers, study coordinators, pharmacists, biostatisticians, quality assurance managers, regulatory affairs managers, and administrative staff among others. Four site readiness practices are included in the research team domain:

1. The research team has sufficient and diverse personnel, to support the roles and functions needed to conduct a clinical trial that is designed to provide a clear answer and enroll trial participants who accurately reflect the population for the disease or condition being studied, with particular consideration for underrepresented and underserved groups.
2. The PI is qualified through experience, training, and mentorship to lead and conduct clinical trials and is free from regulatory debarment and other disciplinary actions that would prevent them from practicing medicine and conducting clinical research.
3. Sub-investigators and other research team members are qualified through experience, training, and mentorship to conduct clinical trials, are well trained in cultural humility and strategies for engaging with underrepresented communities, and are free from disciplinary actions that would prevent them from conducting clinical trials.
4. All research team members receive initial and refresher training to perform clinical trial activities per ICH GCP standards, and as appropriate, have received training that is tailored to an individual’s role and specific to the study protocol.

### Infrastructure

Clinical trial infrastructure consists of the physical and operational software, systems, policies, and facilities necessary for a PI and research team to conduct research. This includes interoperable information systems, equipment, supply management, human resource management, talent development capability, administrative, financial, and legal functions, and business continuity planning. There are 12 site readiness practices that inform sites of the requisite clinical trial infrastructure.

1. Identify all satellite sites, external and community facilities, and contractors utilized to fulfill the requirements of studies.
2. Ensure facilities (including satellite sites, external facilities, and contractors) and equipment are adequate to fulfill the requirements of a study.
3. Provide reliable physical and operational infrastructure (e.g., electric power, internet access, telephone, email, and communications).
4. Along with community affiliates, store documents, materials, product, and equipment in a secure location protected against theft, damage, tampering, or other harms during the duration of a study.
5. Retain study records after the conclusion of a study pursuant to national, state, local, and other applicable requirements and study protocol.
6. Safeguard staff and participants and secure virtual and physical assets (e.g., facilities, records, specimens) during a disruption of operations (e.g., natural disaster).
7. Maintain essential functions after a major disruption of operations (e.g., natural disaster).
8. Protect computers, networks, programs, and data from digital disruptions and attacks.
9. Maintain interoperable information systems and technology capabilities (e.g. data standards, quality control), adequate to support clinical trial conduct.
10. Initiate study (e.g., execute a contract) in a prompt manner.
11. Ensure sufficient processes for hiring and supporting diverse staff to fulfill the roles and functions needed to conduct a clinical trial (e.g., principal/sub-investigators, clinical research associates, research nurses, data managers, and study coordinators).
12. Identify and manage conflicts of interest, including complete financial disclosures for research team members, pursuant to national, state, local, and other applicable requirements and study protocol.

### Study Management

Managing clinical trial protocols requires dedicated personnel, resources, and tools to manage implementation and fulfillment of the study requirements at a site. Study execution should include plans to promote diversity and inclusivity among trial participants, ensure equal access, and establish and sustain trusting relationships with the community. Responsibilities may begin as early as the planning stage before the trial begins, to final participant follow-up and subsequent dissemination of results. Full compliance with the protocol and appropriate levels of oversight and monitoring during the trial ensure that safety and trial integrity are maintained throughout the study, and that there is accurate collection and reporting of data that informs any results. The study management domain comprises nine site readiness practices.

1. Research team utilizes standard operating procedures/processes for the conduct of clinical trials pursuant to national, state, local, and other applicable requirements and study protocol.
2. PI monitors and can demonstrate oversight for all study-related activities, including those functions delegated to satellite sites and contractors, including recruitment, enrollment, and retention suitable for reflecting the diversity of the populations affected by the disease or intervention of study.
3. Research team can execute study initiation, startup, and close-out procedures in a prompt manner.
4. Research team has access to and process for recruiting and retaining eligible study participants, which should include a plan for enrolling adequate numbers of participants from populations that are underrepresented and underserved in clinical trials.
5. Research team can collect, handle, label, store, and ship digital and biological samples (e.g., cultures, blood, serum, plasma, urine, feces, tissues, and imaging) with appropriate documentation pursuant to national, state, local, and other applicable requirements and study protocol.
6. Research team can handle investigational medical products, devices, and other means of intervention safely and securely and can record receipt, expiry, reconstitution, handling, dispensation, transfer, and/or destruction.
7. Research team can establish, maintain, and record calibration for study-specific equipment.
8. Research team can maintain essential study documentation before, during, and after a trial.
9. Responsible party^e^ must report study results to clinicaltrials.gov within the required times before, during, and after the conclusion of a study and has a strategy for dissemination of research findings to stakeholders and participants.

### Data Collection and Management

Data collection and management requires dedicated personnel, resources, and tools that ensure data integrity and lead to generation of high-quality, reliable, and statistically sound data (e.g., statistical design, case report form design and annotation, database design, data entry, data validation, discrepancy management, medical coding, data extraction, and database locking). Five site readiness practices provide guidance on data collection and management:

1. Research team implements controls (e.g., audits, system validations, audit trails, electronic signatures, and documentation) for software and systems involved in processing study-related data pursuant to national, state, local, and other applicable requirements and study protocol.
2. Research team can collect, access, retrieve, and exchange data in a timely, accurate, and complete manner, according to a statistical plan designed to yield definitive responses to study questions.
3. Monitors, sponsor personnel, and regulatory authorities have access to source material, electronic data systems, facilities, and source documents.
4. Research team can ensure quality control of data and source documentation to ensure the integrity and proper reporting of study data.
5. Research team can ensure blinding/masking, while promoting transparency and trust with study participants regarding how their data will be used and who will have access to it.

### Quality Oversight

The outcomes of clinical trials depend greatly on the quality of the data generated. A quality management system, dedicated personnel, and quality monitoring tools are needed to create a culture of quality through process controls and continuous improvement practices, ensuring that research activities are conducted in a manner that complies with regulations, institutional policies, and the study protocol. Three site readiness practices pertain directly to quality oversight:

1. Research team can ensure and verify that the quality requirements have been fulfilled pursuant to national, state, local, and other applicable requirements and study protocol.
2. Research team members are able and empowered to identify, prevent, report, and correct safety and quality issues in a timely fashion.
3. Research team can identify and remediate deficiency findings from regulatory inspections and sponsor audits (e.g., warning letters, FDA Form 483, corrective and preventive action).

### Ethics and Safety

Research teams must protect the rights and welfare of all participants engaged in studies. Human research protection programs promote compliance with relevant legal requirements and ethical standards at all levels to protect trial participants, investigators, and sponsors. Components typically include education and training; quality assurance and compliance; and research review units, including institutional review boards. The ethics and safety domain is composed of seven site readiness practices.

1. Research team can protect the rights and welfare of trial participants pursuant to national, state, local, and other applicable requirements and study protocol.
2. Research team can identify, assess, process, and report safety events (e.g., deviations, malfunctions, deficiencies, and adverse events) pursuant to national, state, local, and other applicable requirements and study protocol.
3. Research team can execute an informed consent/assent process that is respectful of participants and pursuant to national, state, local, and other applicable requirements and study protocol.
4. Research team can maintain confidentiality for study participants, while promoting transparency and trust with participants regarding how their data will be used and who will have access to it.
5. Research team has access to and reports to a properly constituted IRB/ethics committee pursuant to national, state, local, and other applicable requirements and study protocol.
6. Research team engages with study participants, especially vulnerable populations (e.g., children, refugees, people with an intellectual or developmental disability) and populations that have experienced medical abuse and exploitation (e.g., racial and ethnic minorities), in an ethical and culturally appropriate manner, and addresses institutional racism through intentional recruitment and engagement strategies.
7. Research team clearly communicates study risks and benefits to study participants in a manner that is accessible and culturally/linguistically appropriate.

## Discussion

Widespread adoption of the common principles and site readiness practices outlined above could improve the performance and success of clinical trials by streamlining site selection and trial initiation with a common set of expectations for trial sites and sponsors. A shared understanding of the elements for trial site readiness and appropriate documentation offers an opportunity to reduce the duplicative and burdensome site qualifications that delay trials and can provide a foundation for a broader, more efficient, equitable, and quality clinical trials enterprise. A set of metrics should be identified to show progress over time as qualifications are being adopted. An accompanying perspective from the CTSA program provides suggestions of expanding and harmonizing trial site readiness processes relevant to health care systems [17].

Additionally, establishing site readiness practices across sites would represent a first step toward a more comprehensive system of widely accepted qualifications and credentialing. The site readiness practices laid out in this article should first be tested and validated before establishing common qualifications, which could be supported by standardized review processes to create site credentialing. As described in an earlier discussion paper, a harmonized system for gauging site readiness and quality, accepted by the NIH, industry, and other major sponsors, could reduce administrative burden for sites, trial leaders, and sponsors [18]. As qualifications setting and credentialing has improved clinical care in specific areas [19], it can also be used to gain the broad engagement and systematization required to disencumber and improve the international clinical trials enterprise.

### Benefits of Clinical Trial Site Readiness

Buy-in for a shared understanding of site readiness is also a first step for lowering the barrier to entry for clinical trial sites that have not typically participated in the clinical trials enterprise, including community-based practices. Incorporating a diversity of trial sites is crucial for recruiting diverse trial participants, and ultimately for the generalizability of trial results and the ability of trials to inform clinical practice.

Both potential and current sites could use the framework to catalog documentation of site readiness, to appeal to trial sponsors. For sites that are considering participation in clinical trials, the site readiness practices offer a framework, preparing sites for the capabilities and capacity required to conduct a trial. Similarly, the framework clarifies the minimum requirements for operating a trial site and simultaneously describe the complexities inherent in clinical trials. Further consideration of DEIA (diversity, equity, inclusion, and accessibility) issues and the need for enhanced site readiness is provided in an accompanying perspective (Carter-Edwards, Hidalgo B, Lewis-Hall F, Nguyen T, Rutter J. Diversity, equity, inclusion, and access are necessary for clinical trial site readiness. *J Clin Transl Sci.*, in review).

For current sites, the framework can help recruit research team members and engage new collaborators attracted by strong clinical trial programs. The site readiness practices should promote discussions regarding necessary training for research teams, and the role of clinical trial institutions in developing such training. CTSAs are uniquely positioned to lead the development of training and mentorship programs. For example, organizations such as the Society for Clinical Research Sites have established training programs to prepare researchers [20], and the Clinical Trials Transformation Initiative offers recommendations to guide qualification training [21]. Additionally, streamlining training requirements can produce research teams that have the expertise to conduct mentoring, while also providing greater opportunities for staff to focus on clinical trial innovations and refinement of the site readiness practices. They also may encourage greater institution-wide attention and investment in issues for which individual disease-specific investigators may have limited influence, such as contracting and IRB turn-around times. Applying the site readiness practices across individual sites of a multisite trial could reduce protocol deviations, which increase with the number of sites in a trial [22], by improving the standardization of training, infrastructure, and culture at disparate sites.

Trial sponsors, as well as the CROs with which they work, would also benefit from adoption of the site readiness practice framework. Trial sponsors, which typically require compliance with their own qualifications in addition to GCP standards, can use the site readiness practices to compare to their own assessment processes, identifying discordances or specialty requirements, in addition to considering modifying potentially onerous requirements. For example, requirements that site staff are familiar with a sponsor’s publication policy may add little value to the clinical trial. Sponsors that streamline and implement the site readiness practices would have the valuable advantage of more rapid site identification and startup and could build a consistent track record of successful clinical trial programs. The site readiness practices encourage transparency of safety and quality systems and processes, which can promote early dialog between trial site and sponsor regarding necessary safety measures. As trial sites begin to adopt and adapt the site readiness practices, sponsors could access the expanded pool of potential clinical trial sites, including community-based sites, and sites that can accommodate decentralized trials. Moreover, buy-in from trial sites would enable sponsors to build directories of facilities that have adopted the site readiness practices for rapid inclusion in trials.

For trade and membership associations as well as other interest groups, these site readiness practices can facilitate conversations with association members about clinical trial conduct, site qualifications, and their connection to quality and safety. Additionally, as these site readiness practices reflect common elements applicable to all clinical trial sites, they can help promote further consideration for specialized site qualifications. These groups could also use the framework to help develop clinical trial site accreditation processes, in thoughtful coordination with trial sites.

The common principles and site readiness practices elevate person-centeredness in clinical trials and promote responsible and ethical engagement of participants and the general public. The site readiness practices endeavor to embed the tenets of diversity, equity, inclusion, and accessibility into clinical trial sites. These tenets are fundamental to the conduct of clinical trials that generate actionable evidence for use in clinical practice. These site readiness practices also make clinical trial processes and safeguards more transparent to the general public and trial participants. Transparency in these processes can engender greater trust in clinical trials. Similarly, adoption of the site readiness practices provides the public and potential trial participants with greater assurances of quality and safety at the trial site. Trial participants will benefit from a greater understanding of how their personal data is being used in the trial, and from appropriate security measures to protect their data. Ultimately, the site readiness practices aim to reduce the time it takes for drug discoveries to be translated to clinically accepted therapies that people living with disease can access.

### Approaches to Adoption and Implementation

As a next step toward broader uptake of these site readiness practices, organizations should implement, evaluate, and refine the framework with different trial sponsors through a continuous quality improvement process. Organizations should conduct quantitative measurements for trial outcomes^f^ (e.g., duration of recruitment process and number of protocol deviations) and qualitative assessment of the research teams’ experience with the site readiness practices (e.g., practicality of documenting compliance and burden on research team) and disseminate results in the medical literature.

### Call to Action

The wealth of clinical trial experience during the COVID-19 pandemic has shown us that we can execute high-quality, well-designed, definitive clinical trials; and it also showed us that we still need to improve. The starkness of existing inadequacies in the qualifications designed to produce informative, high-quality trials – or the variable implementation of these qualifications – has been made clear and must be addressed. This framework highlights key domains of expectation for organizations to be ready to conduct clinical trials. Supplement A will allow organizations to assess and evaluate their status, take action for addressing inadequacies, and ensure that they are ready for conducting future clinical trials. Adoption of these readiness metrics will usher better, more impactful, and more equitable clinical trials.

## Footnotes

a For more information, see https://www.mmsholdings.com/ (accessed July 5, 2022).

b Working group participants included: Arti Bajpai (Compliance and Quality Integration Consulting); Tracy H. Blumenfeld (RapidTrials); Peter G.Cheryl Gandinetti (Center for Drug Evaluation and Research, FDA); Peter G. Goldschmidt, *co-lead* (Health Improvement Institute); Cheryl Lutz (IQVIA); Douglas Peddicord, *co-lead* (Association of Clinical Research Organizations); Ana Pujols McKee (The Joint Commission); and Peter Vasilenko (Alion HRPP Accreditation Program). Affiliations reflect those at the time the working group met, over the fall of 2013.

c Clinical trial experts included: Anand Mohanish (Pfizer Inc.); Arti Bajpai (Compliance and Quality Integration Consulting); Beverly Brown (Allergan); Ed Connor (I-ACT for Children & George Washington University School of Medicine); Jacqueline Corrigan-Curay (Center for Drug Evaluation and Research, FDA); Federico A. Feldstein (Johnson & Johnson); Grant D. Huang (Department of Veterans Affairs); Anne Marie Inglis (GSK); S. Claiborne Johnston (The University of Texas at Austin); Nya Karanga (Sanofi US); Edward S. Kim (Levine Cancer Institute, Carolinas Healthcare System); Ellen Leschek (National Institute of Diabetes and Digestive and Kidney Diseases, NIH); Freda Lewis-Hall (Pfizer Inc.); Claudia Scala Moy (National Institute of Neurological Disorders and Stroke, NIH); John Oidtman (EMD Serono Inc.); Gail D. Pearson (National Heart, Lung, and Blood Institute, NIH); Jorge Rodriguez-Larrain (Alexion Pharmaceuticals); Matthew T. Roe (Duke Clinical Research Institute); Archana Sah (Genentech, Inc.); Monica Shah (IQVIA); Gina A. Gorgone Simone (Emmes); Reneé Smith (Sarah Cannon Research Institute); and Pamela Tenaerts (Clinical Trials Transformation Institute). Affiliations reflect those at the time the working group considered the site readiness practices, from November 2017 to March 2018.

d The subset of working group participants included S. Claiborne Johnston (Dell Medical School), Arti Bajpai (Compliance and Quality Integration Consulting), Ed Connor (I-ACT for Children & George Washington University School of Medicine), Grant D. Huang (Department of Veterans Affairs), Joseph Pollarine (Johnson & Johnson), Monica Shah (IQVIA), and Gina A. Gorgone Simone (Emmes). Affiliations reflect those at the time the working group met to refine the site readiness practices, in September 2018.

e For more information on who qualifies as a responsible party, see https://www.ecfr.gov/current/title-42/chapter-I/subchapter-A/part-11 (accessed August 15, 2022).

f The CTTI Quality by Design Metrics Framework discusses nine example metrics that clinical trial sites can measure to evaluate the effects of implementing the site readiness practices from this article. https://ctti-clinicaltrials.org/wp-content/uploads/2021/06/CTTI_QbD_Metrics_Framework.pdf (accessed September 8, 2022).

## Supplementary material

For supplementary material accompanying this paper visit https://doi.org/10.1017/cts.2023.541. click here to view supplementary material
