# Supplementary material for: A framework for assessing clinical trial site readiness
Source: J Clin Transl Sci. 2023 May 8;7(1):e151. doi: 10.1017/cts.2023.541 (PMC10346039; doi:10.1017/cts.2023.541)
Supplement: Supplementary file 1 [file ctssup.zip › S2059866123005411sup001.docx]

A Roadmap for Clinical Trial Site Readiness

Supplement B. List of Site Readiness Practices for Clinical Trials, Organized by Domain.

| **Domain** | **Site Readiness Practices** |
| --- | --- |
| Research Team | 1. The research team has sufficient and diverse personnel, to support the roles and functions needed to conduct a clinical trial and enroll trial participants who accurately reflect the patient population for the disease or condition being studied, with particular consideration for underrepresented and underserved groups. 2. The Principal Investigator is qualified through experience, training, and mentorship to lead and conduct clinical trials, and is free from regulatory debarment and other disciplinary actions that would prevent them from practicing medicine and conducting clinical research. 3. Sub-investigators and other research team members are qualified through experience, training, and mentorship to conduct clinical trials, are well trained in cultural humility and strategies for engaging with underrepresented communities, and free from disciplinary actions that would prevent them from conducting clinical trials. 4. All research team members receive initial and refresher training to perform clinical trial activities per ICH GCP standards, and as appropriate, have received training that is tailored to an individual’s role and specific to the study protocol. |
| Infrastructure | 1. Identify all satellite sites, external and community facilities, and contractors utilized to fulfill the requirements of studies. 2. Ensure facilities (including satellite sites, external facilities, and contractors) and equipment are adequate to fulfill the requirements of a study. 3. Provide reliable physical and operational infrastructure (e.g., electric power, internet access, telephone, email, and communications). 4. Along with community affiliates, store documents, materials, product, and equipment in a secure location protected against theft, damage, tampering, or other harms during the duration of a study. 5. Retain study records after the conclusion of a study pursuant to national, state, local, and other applicable requirements and study protocol. 6. Safeguard staff and participants and secure virtual and physical assets (e.g., facilities, records, specimens) during a disruption of operations (e.g., natural disaster). 7. Maintain essential functions after a major disruption of operations (e.g., natural disaster). 8. Protect computers, networks, programs, and data from digital disruptions and attacks. 9. Maintain interoperable information systems and technology capabilities (e.g. data standards, quality control), adequate to support clinical trial conduct. 10. Initiate study (e.g., execute a contract) in a prompt manner. 11. Ensure sufficient processes for hiring and supporting diverse staff to fulfill the roles and functions needed to conduct a clinical trial (e.g., Principal/sub investigators, clinical research associates, research nurses, data managers, and study coordinators). 12. Identify and manage conflicts of interest, including complete financial disclosures for research team members, pursuant to national, state, local, and other applicable requirements and study protocol. |
| Study Management | 1. Research team utilizes standard operating procedures/processes for the conduct of clinical trials pursuant to national, state, local, and other applicable requirements and study protocol. 2. Principal investigator monitors and can demonstrate oversight for all study-related activities, including those functions delegated to satellite sites and contractors, including recruitment, enrollment, and retention suitable for reflecting the diversity of the populations affected by the disease or intervention of study. 3. Research team can execute study initiation, start-up, and close-out procedures in a prompt manner. 4. Research team has access to and process for recruiting and retaining eligible study participants, which should include a plan for enrolling adequate numbers of participants from populations that are underrepresented and underserved in clinical trials. 5. Research team can collect, handle, label, store, and ship digital and biological samples (e.g., cultures, blood, serum, plasma, urine, feces, tissues, imaging) with appropriate documentation pursuant to national, state, local, and other applicable requirements and study protocol. 6. Research team can handle investigational medical products, devices, and other means of intervention safely and securely and can record receipt, expiry, reconstitution, handling, dispensation, transfer, and/or destruction. 7. Research team can establish, maintain, and record calibration for study specific equipment. 8. Research team can maintain essential study documentation before, during, and after a trial. 9. Responsible party^^[[1]](#footnote-1)^^ must report study results to clinicaltrials.gov within the required times before, during, and after the conclusion of a study, and has a strategy for dissemination of research findings to stakeholders and participants. |
| Data Collection and Management | 1. Research team implements controls (e.g., audits, system validations, audit trails, electronic signatures, and documentation) for software and systems involved in processing study-related data pursuant to national, state, local, and other applicable requirements and study protocol. 2. Research team can collect, access, retrieve, and exchange data in a timely, accurate, and complete manner, according to a statistical plan designed to yield definitive responses to study questions. 3. Monitors, sponsor personnel, and regulatory authorities have access to source material, electronic data systems, facilities, and source documents. 4. Research team can ensure quality control of data and source documentation to ensure the integrity and proper reporting of study data. 5. Research team can ensure blinding/masking, while promoting transparency and trust with study participants regarding how their data will be used and who will have access to it. |
| Quality Oversight | 1. Research team can ensure and verify that the quality requirements have been fulfilled pursuant to national, state, local, and other applicable requirements and study protocol. 2. Research team members are able and empowered to identify, prevent, report, and correct safety and quality issues in a timely fashion. 3. Research team can identify and remediate deficiency findings from regulatory inspections and sponsor audits (e.g., warning letters, FDA Form 483, corrective and preventive action). |
| Ethics and Safety | 1. Research team can protect the rights and welfare of trial participants pursuant to national, state, local, and other applicable requirements and study protocol. 2. Research team can identify, assess, process, and report safety events (e.g., deviations, malfunctions, deficiencies, adverse events) pursuant to national, state, local, and other applicable requirements and study protocol. 3. Research team can execute an informed consent/assent process that is respectful of participants and pursuant to national, state, local, and other applicable requirements and study protocol. 4. Research team can maintain confidentiality for study participants, while promoting transparency and trust with participants regarding how their data will be used and who will have access to it. 5. Research team has access to and reports to a properly constituted IRB/ethics committee pursuant to national, state, local, and other applicable requirements and study protocol. 6. Research team engages with study participants, especially vulnerable populations (e.g., children, refugees, people with an intellectual or developmental disability) and populations that have experienced medical abuse and exploitation (e.g., racial and ethnic minorities), in an ethical and culturally appropriate manner, and addresses institutional racism through intentional recruitment and engagement strategies. 7. Research team clearly communicates study risks and benefits to study participants in a manner that is accessible and culturally/linguistically appropriate. |

1. For more information on who qualifies as a responsible party, see <https://www.ecfr.gov/current/title-42/chapter-I/subchapter-A/part-11> (accessed August 15, 2022). [↑](#footnote-ref-1)
