# Supplementary material for: A framework for assessing clinical trial site readiness
Source: J Clin Transl Sci. 2023 May 8;7(1):e151. doi: 10.1017/cts.2023.541 (PMC10346039; doi:10.1017/cts.2023.541)
Supplement: Supplementary file 1 [file ctssup.zip › S2059866123005411sup002.docx]

A Roadmap for Clinical Trial Site Readiness

Supplement A. Guidance and Resources for Domains and Site Readiness Practices

| **Domain 1: Research Team**  Description: Clinical trials are typically led by a principal investigator, who is often a licensed physician or other health care provider with an appropriate clinical background. The principal investigator is the primary individual responsible for the safe and ethical conduct and administration of a clinical trial protocol in compliance with applicable laws and regulations that generates independent, high quality, and reproducible results. Principal investigators may have a research team that contributes to the conduct of a clinical trial at a site. A sub investigator is any individual member of the research team who is supervised by the investigator and delegated to perform critical trial-related procedures and makes important trial-related contributions. In addition, there may be several other individuals who provide administrative or other support to enable trial activities to occur at a site. Examples of individuals who may help with conducting a clinical trial include research nurse, pharmacist, coordinator, data manager, biostatistician, quality assurance manager, or regulatory affairs manager. | | |
| --- | --- | --- |
| **Site Readiness Practices** | **Guidance and Resources** | **Potential Documentation** |
| 1. The institution has sufficient and diverse personnel, to support the roles and functions needed to conduct a clinical trial and enroll trial participants who accurately reflect the patient population for the disease or condition being studied, with particular consideration for underrepresented and underserved groups. | - National Cancer Institute (NCI) – [Research Team Members](https://www.cancer.gov/about-cancer/treatment/clinical-trials/what-are-trials/team)   *Describes the roles and functions of clinical trial personnel.*   - Society for Clinical Research Sites (SCRS) – [Diversity Site Assessment Tool](https://myscrs.org/dsat/)   *Evaluates capacity to accommodate needs of diverse trial participants via self-assessment tool.*   - Multi-Regional Clinical Trials – [Workforce and Diversity: Training and Development](https://mrctcenter.org/diversity-in-clinical-research/tools/toolkit/#1608087659178-a0df9733-d663)   *Outlines potential activities, outputs, and outcomes for development of a diverse workforce.* | - Organizational charts - Documentation of study roles and responsibilities - Job descriptions - Hiring timeline and procedures |
| 1. The Principal Investigator is qualified through experience, training, and mentorship to lead and conduct clinical trials, and is free from regulatory debarment and other disciplinary actions that would prevent them from practicing medicine and conducting clinical trials. | - Association of Clinical Research Professionals (ACRP) – [Certified Principal Investigator](https://www.acrpnet.org/certifications/pi-certification/)   *Certifies proficiency in knowledge and skills required of principal investigator.*   - Clinical Trials Transformation Initiative (CTTI) – [Investigator Qualification](https://www.ctti-clinicaltrials.org/projects/investigator-qualification)   *Provides recommendations and resources for recognizing experience and identifying knowledge gaps for investigators.*   - 21 CFR Parts 54 and 312 (subpart D)   *Outlines the general responsibilities of clinical trial sponsors and investigators.*   - TransCelerate – [Mutual Recognition Program for IHC E6 GCP Training](https://transcelerate-gcp-mutual-recognition.com/home)   *Offers Good Clinical Practice training for investigators.*   - CTTI – [Recommendations for Strengthening the Investigator Site Community](https://ctti-clinicaltrials.org/wp-content/uploads/2021/06/CTTI_Investigator_Community_Recs.pdf)   *Provides recommendations for reducing investigator turnover.* | - Current and valid medical license - Up-to-date documentation of education and clinical research experience (e.g., CVs, certifications) |
| 1. Sub-investigators and other research team members are qualified through experience, training, and mentorship to conduct clinical trials, are well trained in cultural humility and strategies for engaging with underrepresented communities, and free from disciplinary actions that would prevent them from conducting clinical trials. | ACRP –   - [Project Manager Subspeciality Designation](https://acrpnet.org/certifications/acrp-pm/)   *Certifies project management proficiency in clinical research professionals.*   - [Clinical Research Associate Certification](https://www.acrpnet.org/certifications/cra-certification/)   *Certifies proficiency in monitoring and supervising conduct of clinical trials.*   - [Clinical Research Coordinator Certification](https://www.acrpnet.org/certifications/crc-certification/)   *Certifies proficiency in coordinating and facilitating clinical trial activities compliant with GCP.*   - [Certified Professional](https://www.acrpnet.org/certifications/acrp-cp-certification/)   *Certifies proficiency of clinical research professionals of any role.* | - Up-to-date documentation of education and clinical research experience (e.g., CVs, certifications) |
| 1. All research team members receive initial and refresher training to perform clinical trial activities per ICH GCP standards, and as appropriate, have received training that is tailored to an individual’s role and specific to the study protocol. | - ACRP – [Core Competency Guidelines for Clinical Research Coordinators](https://www.acrpnet.org/core-competency-guidelines-clinical-research-coordinators-crcs/)   *Details basic competencies required of clinical research coordinators.*   - ACRP – [Competency Domains for Clinical Research Professionals](https://acrpnet.org/competency-domains-clinical-research-professionals/)   *Describes competencies across 8 domains that are required of clinical research professionals.*   - TransCelerate – [Site Qualification and Training Solutions](http://www.transceleratebiopharmainc.com/assets/site-qualification-and-training/)   *Provides information regarding mutual recognition of GCP and electronic data capture system training.*   - Sponsor-provided ICH GCP training   *Many trial sponsors provide their own GCP training.*   - DIA – [Certificate Program](https://www.diaglobal.org/en/course-listing/certificate-program)   *Offers certification in safety and pharmacovigilance and patient engagement.*   - CTTI – [Recommendations: Identifying Qualified Investigators and Their Delegates to Conduct Sponsored Clinical Trials](https://www.ctti-clinicaltrials.org/wp-content/uploads/2021/06/CTTI_Investigator_Qualification_Recs.pdf)   *Provides recommendations for the preparation and qualification of investigators and their delegates.*   - CTTI – [Recommendations: Good Clinical Practice (GCP) Training for Investigators](https://ctti-clinicaltrials.org/wp-content/uploads/2021/06/CTTI_GCP_Recs.pdf)   *Provides recommendations for completing GCP training for investigators.* | - Adopted core competencies - Workforce development plans and curriculums - Up-to-date documentation of training for all site personnel - Policies and procedures for the periodic review of site personnel training/qualifications against current duties |

| **Domain 2: Infrastructure**  Description: Clinical trial infrastructure consists of the physical and operational software, systems, policies, and facilities necessary for a principal investigator and research team to conduct research. This includes information systems, equipment, supply management, human resource management, talent development capability, administrative, financial, and legal functions, and business continuity planning. | | |
| --- | --- | --- |
| **Site Readiness Practices** | **Guidance and Resources** | **Potential Documentation** |
| 1. Identify all satellite sites, external and community facilities, and contractors utilized to fulfill the requirements of studies. | - 21 CFR 312 Subpart D   *Outlines the general responsibilities of clinical trial sponsors and investigators.*   - FDA – [Guidance for Industry: Investigator Responsibilities — Protecting the Rights, Safety, and Welfare of Study Subjects](https://www.fda.gov/downloads/Drugs/GuidanceComplianceRegulatoryInformation/Guidances/UCM187772.pdf)   *Provides overview of responsibilities of investigator regarding trial oversight and human subjects protection.*   - FDA – [Frequently Asked Questions – Statement of Investigator (Form FDA 1572)](https://www.fda.gov/media/78830/download)   *Describes how to complete Statement of Investigator.*   - FDA – [Instructions for filling out FDA Form 1572 – Statement of Investigator](https://www.fda.gov/media/79326/download)   *Provides instructions on completing Statement of Investigator.*   - CTTI – [Investigator Community: Recommendations for Developing Site-Based Research Infrastructure & Staff](https://ctti-clinicaltrials.org/wp-content/uploads/2021/06/CTTI_Investigator_Community_Recs.pdf)   *Provides recommendations for hiring, training, and supporting clinical trial staff.* | - List/description |
| 1. Ensure facilities (including satellite sites, external facilities, and contractors) and equipment are adequate to fulfill the requirements of a study. | - 21 FR 312 Subpart D   *Outlines the general responsibilities of clinical trial sponsors and investigators.*   - FDA – [Guidance for Industry: Investigator Responsibilities — Protecting the Rights, Safety, and Welfare of Study Subjects](https://www.fda.gov/downloads/Drugs/GuidanceComplianceRegulatoryInformation/Guidances/UCM187772.pdf)   *Provides overview of responsibilities of investigator regarding trial oversight and human subjects protection.*   - CTTI – [Investigator Community: Recommendations for Optimizing Trial Execution and Conduct](https://ctti-clinicaltrials.org/wp-content/uploads/2021/06/CTTI_Investigator_Community_Recs.pdf)   *Provides recommendations for successfully preparing for the conduct of a clinical trial.*   - ICH E6(R2): Guideline for Good Clinical Practice – [Section 8: Essential Documents for the Conduct of a Clinical Trial](https://ichgcp.net/8-essential-documents-for-the-conduct-of-a-clinical-trial)   *Describes the documents essential to good clinical practice within clinical trials.* | - List of equipment and hardware |
| 1. Provide reliable physical and operational infrastructure (e.g., electric power, internet access, telephone, email, and communications). | - 21 CFR 312 Subpart D   *Outlines the general responsibilities of clinical trial sponsors and investigators.*   - FDA – [Guidance for Industry: Investigator Responsibilities — Protecting the Rights, Safety, and Welfare of Study Subjects](https://www.fda.gov/downloads/Drugs/GuidanceComplianceRegulatoryInformation/Guidances/UCM187772.pdf)   *Provides overview of responsibilities of investigator regarding trial oversight and human subjects protection.*   - ICH E6(R2): Guideline for Good Clinical Practice – [Section 8: Essential Documents for the Conduct of a Clinical Trial](https://ichgcp.net/8-essential-documents-for-the-conduct-of-a-clinical-trial)   *Describes the documents essential to good clinical practice within clinical trials.* | - List/description |
| 1. Along with community affiliates, store documents, materials, product, and equipment in a secure location protected against theft, damage, tampering, or other harms during the duration of a study. | - American Society of Health-System Pharmacists – [Guidelines for the Management of Investigational Drug Products](https://www.ashp.org/-/media/assets/policy-guidelines/docs/guidelines/management-investigational-drug-products.ashx)   *Provides guidelines for managing investigational drug products throughout the course of a clinical trial.* | - Policies and procedures - List/description |
| 1. Retain study records after the conclusion of a study pursuant to national, state, local, and other applicable requirements and study protocol. | - National Institute of Allergy and Infectious Diseases – [Policy on Storage and Retention of Clinical Research Records](https://www.niaid.nih.gov/sites/default/files/StorageRetentionClinicalResearchRecordsPolicyFinal.pdf)   *Outlines policy for retaining clinical research records.*   - Division of Microbiology and Infectious Diseases – [Source Documentation Standards](https://www.dmidcroms.com/Shared%20Documents/Source%20Documentation%20Standards_English.pdf)   *Outlines standards for the documentation and retention of source data in clinical trials.*   - FDA – [Guidance for Sponsors, Clinical Investigators, and IRBs: Data Retention when Subjects Withdraw from FDA-Regulated Clinical Trials](https://www.fda.gov/regulatory-information/search-fda-guidance-documents/data-retention-when-subjects-withdraw-fda-regulated-clinical-trials)   *Provides guidance on retaining data after an individual ceases participation in a clinical trial.* | - Documentation of archival capabilities |
| 1. Safeguard staff and participants and secure virtual and physical assets (e.g., facilities, records, specimens) during a disruption of operations (e.g., natural disaster). | - Association of Academic Health Centers – [Managing Emergency Preparedness](http://www.aahcdc.org/Portals/41/Publications-Resources/BooksAndReports/AAHC_Emergency_Prep_08.pdf)   *Provides guidance to academic health centers on managing emergency response activities.*   - NIH – [Design Requirements Manual](https://orf.od.nih.gov/TechnicalResources/Documents/DRM/DRM1.503262020.pdf)   *Outlines designs and technical criteria for buildings and facilities where clinical trials take place.*   - National Academies of Sciences, Engineering, and Medicine (NASEM) – [Strengthening Disaster Resilience of the Academic Biomedical Research Community](https://nap.nationalacademies.org/resource/24827/081010_Academic_%20Resilience_%20recs.pdf)   *Provides recommendations on enhancing the disaster resilience of clinical research sites and teams.* | - Emergency response plan |
| 1. Maintain essential functions after a major disruption of operations (e.g., natural disaster). | - NASEM – [Strengthening Disaster Resilience of the Academic Biomedical Research Community](https://nap.nationalacademies.org/resource/24827/081010_Academic_%20Resilience_%20recs.pdf)   *Provides recommendations on enhancing the disaster resilience of clinical research sites and teams.*   - International Organization for Standardization – [ISO 22301 – Business Continuity Management System Requirements](https://www.iso.org/standard/50038.html)   *Describes requirements of a documented management system for disruptive incidents*. | - Business continuity plan |
| 1. Protect computers, networks, programs, and data from digital disruptions and attacks. | - National Institute of Standards and Technology – [Cybersecurity Framework](https://www.nist.gov/cyberframework)   *Provides a framework to protect critical infrastructure from cyber risks.* | - Cybersecurity plan |
| 1. Maintain interoperable information systems and technology capabilities (e.g. data standards, quality control), adequate to support clinical trial conduct. | - 21 CFR Part 11   *Describes FDA regulations for electronic records and signatures.*   - FDA – [Guidance for Industry: Electronic Source Data in Clinical Investigations](https://www.fda.gov/regulatory-information/search-fda-guidance-documents/electronic-source-data-clinical-investigations)   *Provides guidance on the capture, review, and retention of electronic source data.* | - Contractor or on-site IT department |
| 1. Initiate study (e.g., execute a contract) in a prompt manner. | - CTTI – [Recommendations for Strengthening the Investigator Site Community](https://ctti-clinicaltrials.org/wp-content/uploads/2021/06/CTTI_Investigator_Community_Recs.pdf): III. Recommendations for Site Budget and Contract Negotiations   *Provides recommendations on budget development and contract negotiations for sponsors and trial sites.*   - ICH E6(R2): Guideline for Good Clinical Practice – [Section 8: Essential Documents for the Conduct of a Clinical Trial](https://ichgcp.net/8-essential-documents-for-the-conduct-of-a-clinical-trial)   *Describes the documents essential to good clinical practice within clinical trials.*   - Accelerated Research Agreements – [Accelerated Research Agreements Initiative](https://ara4us.org/)   *Provides broadly acceptable templates for agreements that may accelerate study initiation.* | - Policies and procedures - Access to attorney - Sign off protocol and timeline |
| 1. Ensure sufficient processes for hiring and supporting diverse staff to fulfill the roles and functions needed to conduct a clinical trial (e.g., Principal/sub investigators, clinical research associates, research nurses, data managers, and study coordinators). | - ACRP – [Hiring Guidelines for Entry Level Clinical Research Coordinators](https://acrpnet.org/acrp-partners-advancing-the-clinical-research-workforce/hiring-guidelines-for-entry-level-clinical-research-coordinators/)   *Outlines competency-based guidelines for hiring entry level clinical research coordinators.*   - SCRS – [Diversity Site Assessment Tool](https://myscrs.org/dsat/)   *Evaluates capacity to accommodate needs of diverse trial participants via self-assessment tool.* | - Documentation of study roles and responsibilities - Job descriptions - Hiring timeline and procedures |
| 1. Identify and manage conflicts of interest, including complete financial disclosures for research team members, pursuant to national, state, local, and other applicable requirements and study protocol. | - 21 CFR 54   *Describes regulations governing financial disclosures for clinical investigators.*   - FDA – [Guidance for Clinical Investigators, Industry and FDA Staff: Financial Disclosure by Clinical Investigators](https://www.fda.gov/downloads/RegulatoryInformation/Guidances/UCM341008.pdf)   *Outlines FDA guidance on regulatory compliance with financial disclosures.* | - Policies and procedures |

| **Domain 3: Study Management** Description: Managing clinical trial protocols requires dedicated personnel, resources, and tools to manage implementation and fulfillment of the study requirements at a site. Responsibilities may begin as early as the planning stage before the trial begins all the way through to final participant follow-up and subsequent dissemination of results. Full compliance with the protocol and appropriate levels of oversight and monitoring during the trial are needed in order to ensure that safety is maintained throughout the study, integrity of the study is maintained and that there is accurate collection and reporting of data that leads to any results at its conclusion. | | |
| --- | --- | --- |
| **Site Readiness Practices** | **Guidance and Resources** | **Potential Documentation** |
| 1. Research team utilizes standard operating procedures/processes for the conduct of clinical trials pursuant to national, state, local, and other applicable requirements and study protocol. | - FDA – [Guidance for Industry: Oversight of Clinical Investigations – A Risk-based Approach to Monitoring](https://www.fda.gov/regulatory-information/search-fda-guidance-documents/oversight-clinical-investigations-risk-based-approach-monitoring)   *Outlines FDA guidance for developing risk-based monitoring strategies for clinical investigations.*   - TransCelerate & SCRS – [Informational Programs for Site Staff Conducting Clinical Research](http://myscrs.org/learningcampus/site-management-modules/)   *Reviews Principal Investigator oversight and GCP practices through video modules.*   - TransCelerate – [Elements of a Clinical Quality Management System Conceptual Framework](https://www.transceleratebiopharmainc.com/assets/quality-management-system-solutions/)   *Provides resources for institutions to build and maintain a clinical quality management system.* | - Process and procedures - Error correction procedures and documentations- CAPA - Past audits and inspections, if applicable |
| 1. Principal investigator monitors and can demonstrate oversight for all study-related activities, including those functions delegated to satellite sites and contractors, including recruitment, enrollment, and retention suitable for reflecting the diversity of the populations affected by the disease or intervention of study. | - FDA – [Guidance for Industry: Investigator Responsibilities – Protecting the Rights, Safety and Welfare of Study Subjects](https://www.fda.gov/regulatory-information/search-fda-guidance-documents/investigator-responsibilities-protecting-rights-safety-and-welfare-study-subjects)   *Provides overview of responsibilities of investigator regarding trial oversight and human subjects protection.*   - 21 CFR 312 Subpart D   *Outlines the general responsibilities of clinical trial sponsors and investigators.*   - FDA – [Guidance for Industry: Oversight of Clinical Investigations – A Risk-Based Approach to Monitoring](https://www.fda.gov/regulatory-information/search-fda-guidance-documents/oversight-clinical-investigations-risk-based-approach-monitoring)   *Outlines FDA guidance for developing risk-based monitoring strategies for clinical investigations.*   - TransCelerate & SCRS – [Informational Programs for Site Staff Conducting Clinical Research](http://myscrs.org/learningcampus/site-management-modules/)   *Reviews Principal Investigator oversight and GCP practices through video modules.* | - Process and procedures supporting oversight steps - Quality management processes and system of communications - Organizational structure for delegation of responsibilities - Oversight documentations |
| 1. Research team can execute study initiation, start-up, and close-out procedures in a prompt manner. | - TransCelerate – [Clinical Content & Reuse Solutions](http://www.transceleratebiopharmainc.com/assets/common-protocol-template/)   *Provides resources and tools for common content to be reused across clinical trial documents.*   - TransCelerate – [Site Qualification and Training Solutions](http://www.transceleratebiopharmainc.com/assets/site-qualification-and-training/)   *Provides information regarding mutual recognition of GCP and electronic data capture system training.*  CTTI – [Recommendations: Identifying Qualified Investigators and Their Delegates to Conduct Sponsored Clinical Trials](https://www.ctti-clinicaltrials.org/wp-content/uploads/2021/06/CTTI_Investigator_Qualification_Recs.pdf)  *Provides recommendations for the preparation and qualification of investigators and their delegates.* | - Showing the quality and promptness of turning these documents around - Project management plans from previous studies, if applicable - Policies and procedures for disposition of study supplies, any equipment provided, and investigational product |
| 1. Research team has access to and process for recruiting and retaining eligible study participants, which should include a plan for enrolling adequate numbers of participants from populations that are underrepresented and underserved in clinical trials. | - CTTI – [Planning for Successful Trial Recruitment](https://ctti-clinicaltrials.org/wp-content/uploads/2021/06/CTTI_Recruitment_Recs.pdf)   *Provides recommendations and framework for improving trial participant recruitment.*   - FDA – [Guidance for Industry: Diversity Plans to Improve Enrollment of Participants From Underrepresented Racial and Ethnic Populations in Clinical Trials](https://www.fda.gov/regulatory-information/search-fda-guidance-documents/diversity-plans-improve-enrollment-participants-underrepresented-racial-and-ethnic-populations)   *Provides FDA recommendations for developing racial and ethnic diversity plans for clinical trials.* | - Policies and procedures - Record of previous performance, if applicable |
| 1. Research team can collect, handle, label, store, and ship digital and biological samples (e.g., cultures, blood, serum, plasma, urine, feces, tissues, imaging) with appropriate documentation pursuant to national, state, local, and other applicable requirements and study protocol. | - NCI – [Biospecimen Collection, Processing, Storage, Retrieval, and Dissemination](https://biospecimens.cancer.gov/bestpractices/to/bcpsrd.asp)   *Provides resources on the collection and handling of biospecimens.*   - International Air Transport Association – [Dangerous Goods Regulations](http://www.iata.org/publications/dgr/pages/index.aspx) for UN 3373, Biological Substance, Category B, and Packing Instruction 650 and UN 2814 Category A Infectious Substances   *Outlines specific international dangerous goods regulations relevant to clinical trials.*   - U.S. Department of Transportation – [Transporting Infectious Substances Safely](https://www.phmsa.dot.gov/sites/phmsa.dot.gov/files/2020-04/Transporting-Infectious-Substances-Safely.pdf)   *Provides guidance on regulatory compliance with infectious substance transportation.*   - 42 CFR 71.54   *Describes federal regulations on the importation of infectious substances.* | - Policies and procedures |
| 1. Research team can handle investigational medical products, devices, and other means of intervention safely and securely and can record receipt, expiry, reconstitution, handling, dispensation, transfer, and/or destruction. | - ASHP – [Guidelines for the Management of Investigational Drug Products](https://www.ashp.org/-/media/assets/policy-guidelines/docs/guidelines/management-investigational-drug-products.ashx?la=en&hash=51D88DFB7BF0F706667BE563295F9E5A6FAE8C85)   *Provides guidelines for managing investigational drug products throughout the course of a clinical trial.*   - ICH GCP 5.14 – [Supplying and Handling Investigational Product(s)](https://ichgcp.net/5-sponsor)   *Provides GCP guidelines for handling investigational products.* | - Policies and procedures - IMP storage and accountability logs and records |
| 1. Research team can establish, maintain, and record calibration for study specific equipment. | - CMS – [Clinical Laboratory Improvement Amendments of 1988 Regulations](https://www.cms.gov/Regulations-and-Guidance/Legislation/CLIA/index.html)   *Provides resources on clinical laboratory testing regulations.* | - Policies and procedures - Maintenance records |
| 1. Research team can maintain essential study documentation before, during, and after a trial. | - ICH E6(R2): Guideline for Good Clinical Practice – [Section 8: Essential Documents for the Conduct of a Clinical Trial](https://ichgcp.net/8-essential-documents-for-the-conduct-of-a-clinical-trial)   *Describes the documents essential to good clinical practice within clinical trials.* | - Policies and procedures - Audit and inspection results |
| 1. Responsible party must report study results to clinicaltrials.gov within the required times before, during, and after the conclusion of a study, and has a strategy for dissemination of research findings to stakeholders and participants. | - CONSORT – [Standards for Reporting](https://bmcmedicine.biomedcentral.com/articles/10.1186/1741-7015-8-18)   *Offers guidelines for reporting randomized clinical trial data.*   - International Committee of Medical Journal Editors – [Recommendations for the Conduct, Reporting, Editing, and Publication of Scholarly work in Medical Journals](http://www.icmje.org/icmje-recommendations.pdf)   *Provides recommendations for reporting on clinical trials in medical journals.* | - Policies and procedures - Record of previous performance, if applicable |

| **Domain 4: Data Collection & Management**  Description: Clinical trials are intended to generate data for proving or disproving a hypothesis, including demonstration of efficacy and safety of medical products. Data collection and management requires dedicated personnel, resources, and tools that ensure data integrity and lead to generation of high-quality, reliable, and statistically sound data (e.g., case report form design and annotation, database designing, data-entry, data validation, discrepancy management, medical coding, data extraction, and database locking). | | |
| --- | --- | --- |
| **Site Readiness Practices** | **Guidance and Resources** | **Potential Documentation** |
| 1. Research team implements controls (e.g., audits, system validations, audit trails, electronic signatures, and documentation) for software and systems involved in processing study-related data pursuant to national, state, local, and other applicable requirements and study protocol. | - Society for Clinical Data Management – [Good Clinical Data Management Practices](http://www.scdm.org/publications/gcdmp/)   *Provides guidance on implementing clinical data management processes.*   - 21 CFR Part 11   *Describes FDA regulations for electronic records and signatures.*   - FDA – [Guidance for Industry: Electronic Records; Electronic Signatures – Scope and Application](https://www.fda.gov/regulatory-information/search-fda-guidance-documents/part-11-electronic-records-electronic-signatures-scope-and-application)   *Describes FDA guidance on regulations for electronic records and signatures.*   - FDA – [Guidance for Industry: Computerized Systems Used in Clinical Investigations](https://www.fda.gov/regulatory-information/search-fda-guidance-documents/computerized-systems-used-clinical-investigations)   *Provides FDA guidance on the use of computerized systems in clinical investigations.*   - FDA – [Guidance for Industry: Electronic Source Data in Clinical Investigations](https://www.fda.gov/downloads/Drugs/GuidanceComplianceRegulatoryInformation/Guidances/UCM328691.pdf)   *Provides guidance on the capture, review, and retention of electronic source data.* | - System specifications |
| 1. Research team can collect, access, retrieve, and exchange data in a timely, accurate, and complete manner, according to a statistical plan designed to yield definitive responses to study questions. | - Clinical Data Interchange Standards Consortium (CDISC) – [Standards in the Clinical Research Process](https://www.cdisc.org/standards)   *Outlines foundational standards for clinical trial data collection, organization, and analysis.*   - CDISC – [Therapeutic Area User Guides](https://www.cdisc.org/standards/therapeutic-areas)   *Details the extension of CDISC foundational standards to specific therapeutic areas.* | - Policies and procedures - Infrastructure - Process - Quality assurance - Resources - Personnel |
| 1. Monitors, sponsor personnel, and regulatory authorities have access to source material, electronic data systems, facilities, and source documents. | - FDA – [Guidance for Industry: Oversight of Clinical Investigations – A Risk-Based Approach to Monitoring](https://www.fda.gov/regulatory-information/search-fda-guidance-documents/oversight-clinical-investigations-risk-based-approach-monitoring)   *Outlines FDA guidance for developing risk-based monitoring strategies for clinical investigations.* | - Policies and procedures |
| 1. Research team can ensure quality control of data and source documentation to ensure the integrity and proper reporting of study data. | - FDA – [Guidance for Industry: Data Integrity and Compliance With CGMP](https://www.fda.gov/downloads/drugs/guidancecomplianceregulatoryinformation/guidances/ucm495891.pdf)   *Describes FDA guidance on the role of data integrity in GMP for drugs and biologics.* | - Policies and procedures to assure information is attributable, legible, contemporaneous, original, and accurate - Limited access record storage area |
| 1. Research team can ensure blinding/masking, while promoting transparency and trust with study participants regarding how their data will be used and who will have access to it. | - PHUSE – [Protection of Personal Data in Clinical Documents – A Model Approach](https://phuse.s3.eu-central-1.amazonaws.com/Deliverables/Data+Transparency/Protection+of+Personal+Data+in+Clinical+Documents+A+Model+Approach.pdf)   *Outlines anonymization strategies to protect personal data through clinical trial data sharing.*   - PHUSE – [De-identification and Anonymization of Individual Patient Data in Clinical Studies – A Model Approach](https://phuse.s3.eu-central-1.amazonaws.com/Deliverables/Data+Transparency/De-identification+and+Anonymization+of+Individual+Patient+Data+in+Clinical+Studies+a+Model+Approach.pdf)   *Outlines model to de-identify and anonymize personal data in clinical trials.* | - Policies and procedures for blinding/unblinding - Availability of independent staff person who can have unblinded duties only - Adequate infrastructure |

| **Domain 5: Quality Oversight** Description: The outcomes of clinical trials depend greatly on the quality of the data generated. A quality management system, dedicated personnel, and quality monitoring tools are needed to create a culture of quality through process controls and continuous improvement practices to ensure that research activities are conducted in a manner that complies with regulations, institutional policies, and the study protocol. | | |
| --- | --- | --- |
| **Site Readiness Practices** | **Guidance and Resources** | **Potential Documentation** |
| 1. Research team can ensure and verify that the quality requirements have been fulfilled pursuant to national, state, local, and other applicable requirements and study protocol. | - CTTI – [Recommendations for Efficient and Effective Monitoring as a Component of Quality Assurance in the Conduct of Clinical Trials](https://ctti-clinicaltrials.org/wp-content/uploads/2021/06/CTTI_Monitoring_Recs.pdf)   *Provides recommendations for clinical trial monitoring to promote and improve quality.*   - FDA – [Guidance for Industry: Oversight of Clinical Investigations — A Risk-Based Approach to Monitoring](https://www.fda.gov/downloads/Drugs/GuidanceComplianceRegulatoryInformation/Guidances/UCM269919.pdf)   *Outlines FDA guidance for developing risk-based monitoring strategies for clinical investigations.*   - CTTI – [Enhancing Clinical Evidence by Proactively Building Quality into Clinical Trials](https://journals.sagepub.com/doi/pdf/10.1177/1740774516643491)   *Describes CTTI quality-by-design principles and recommendations.* | - Policies and procedures - Process controls - Past performance, if applicable |
| 1. Research team members are able and empowered to identify, prevent, report, and correct safety and quality issues in a timely fashion. | - FDA – [Guidance for Industry: Q9 Quality Risk Management](https://www.fda.gov/downloads/Drugs/Guidances/ucm073511.pdf)   *Outlines tools for quality risk management for the pharmaceutical industry.*   - CTTI – [Enhancing Clinical Evidence by Proactively Building Quality into Clinical Trials](https://journals.sagepub.com/doi/pdf/10.1177/1740774516643491)   *Describes CTTI quality-by-design principles and recommendations.*   - TransCelerate – [Elements of a Clinical Quality Management System Conceptual Framework](https://www.transceleratebiopharmainc.com/assets/quality-management-system-solutions/)   *Provides resources for institutions to build and maintain a clinical quality management system.*   - EMA – [Reflection Paper on Risk Based Quality Management in Clinical Trials](https://www.ema.europa.eu/documents/scientific-guideline/reflection-paper-risk-based-quality-management-clinical-trials_en-0.pdf)   *Reviews development of risk-based approach to clinical trial quality management.*   - ACRP – [Site Quality Management Tools](https://www.acrpnet.org/courses/site-quality-management-tools-sops-metrics-training/)   *Reviews quality management and improvement processes for clinical trials.* |  |
| 1. Research team can identify and remediate deficiency findings from regulatory inspections and sponsor audits (e.g., warning letters, FDA Form 483, corrective and preventive action). | - TransCelerate – [Elements of a Clinical Quality Management System Conceptual Framework](https://www.transceleratebiopharmainc.com/assets/quality-management-system-solutions/)   *Provides resources for institutions to build and maintain a clinical quality management system.*   - FDA – [Bioresearch Monitoring: Clinical Investigators and Sponsor Investigators](https://www.fda.gov/downloads/ICECI/EnforcementActions/BioresearchMonitoring/ucm133773.pdf)   *Describes the FDA Bioresearch Monitoring compliance programs.*   - FDA – [Corrective and Preventative Action Basics](https://www.fda.gov/downloads/training/cdrhlearn/ucm421767.pdf)   *Reviews the role and requirements of Corrective and Preventive Action.*   - FDA – [Guidance for Industry: Q10 Pharmaceutical Quality System](https://www.fda.gov/regulatory-information/search-fda-guidance-documents/q10-pharmaceutical-quality-system)   *Describes a model for a quality management system for the pharmaceutical industry.* | - Policies and procedures - Past performance, if applicable - Resources and personnel |

| **Domain 6: Ethics & Safety** Description: Research teams must protect the rights and welfare of human subjects participating in studies. Human research protection programs promote compliance with relevant legal requirements and ethical standards at all levels to protect human subjects, investigators, sponsors, and research participants. Components typically include education and training; quality assurance and compliance; and research review units, including institutional review boards. | | |
| --- | --- | --- |
| **Site Readiness Practices** | **Guidance and Resources** | **Potential Documentation** |
| 1. Research team can protect the rights and welfare of research participants pursuant to national, state, local, and other applicable requirements and study protocol. | - Association for the Accreditation of Human Research Protection Programs – [Accreditation Procedures](http://aahrpp.org/accreditation/get-accredited/overview)   *Details process for accreditation of quality human research protection program.*   - FDA – [Guidance for Industry Investigator Responsibilities — Protecting the Rights, Safety, and Welfare of Study Subjects](https://www.fda.gov/downloads/Drugs/GuidanceComplianceRegulatoryInformation/Guidances/UCM187772.pdf)   *Provides overview of responsibilities of investigator regarding trial oversight and human subjects protection.* | - Policies and procedures |
| 1. Research team can identify, assess, process, and report safety events (e.g., deviations, malfunctions, deficiencies, adverse events) pursuant to national, state, local, and other applicable requirements and study protocol. | - CTTI – [Recommendations for IND Safety and Communication](https://ctti-clinicaltrials.org/wp-content/uploads/2021/06/CTTI_IND_Safety_Recs.pdf)   *Provides recommendations for development and implementation of safety assessments.*   - CTTI – [Improving Reporting of Unexpected Serious Adverse Events to Investigational New Drug Investigators](https://ctti-clinicaltrials.org/wp-content/uploads/2021/06/CTTI_SAE_Reporting_Recs.pdf)   *Provides recommendations on improving the usefulness of safety reports.* | - Policies and procedures - Past performance, if applicable - Evidence of adequate training |
| 1. Research team can execute an informed consent/assent process that is respectful of participants and pursuant to national, state, local, and other applicable requirements and study protocol. | - ICH GCP 4.8 – [Informed Consent of Trial Subjects](https://ichgcp.net/4-investigator)   *Provides GCP guidelines for conducting informed consents of clinical trial participants.*   - FDA – [Guidance for IRBs and Clinical Investigators: A Guide to Informed Consent - Information Sheet](https://www.fda.gov/RegulatoryInformation/Guidances/ucm126431.htm)   *Outlines FDA guidance on conducting and documenting informed consents.*   - CTTI – [Recommendations for Informed Consent](https://ctti-clinicaltrials.org/wp-content/uploads/2021/06/CTTI_Informed_Consent_Recs.pdf)   *Provides recommendations for improving the informed consent process.* | - Policies and procedures |
| 1. Research team can maintain confidentiality for study participants, while promoting transparency and trust with participants regarding how their data will be used and who will have access to it. | - [Clinical Research and the HIPAA Privacy Rule](https://privacyruleandresearch.nih.gov/clin_research.asp)   *Describes the implications of the HIPAA Privacy Rule for clinical trial activities.* | - Policies and procedures - Adequate infrastructure |
| 1. Research team has access to and reports to a properly constituted IRB/ethics committee pursuant to national, state, local, and other applicable requirements and study protocol. | - FDA – [Guidance for Clinical Investigators, Sponsors, and IRBs: Adverse Event Reporting to IRBs — Improving Human Subject Protection](https://www.fda.gov/regulatory-information/search-fda-guidance-documents/adverse-event-reporting-irbs-improving-human-subject-protection)   *Details FDA guidance for reporting unanticipated problems in clinical trials to IRBs.* | - IRB/IEC written policies, procedures, and timelines for reviewing and approving new research |
| 1. Research team engages with study participants, especially vulnerable populations (e.g., children, refugees, people with an intellectual or developmental disability) and populations that have experienced medical abuse and exploitation (e.g., racial and ethnic minorities), in an ethical and culturally appropriate manner, and addresses institutional racism through intentional recruitment and engagement strategies. | - FDA – [Guidance for Industry and FDA Staff: Evaluation and Reporting of Age-, Race-, and Ethnicity-Specific Data in Medical Device Clinical Studies](https://www.fda.gov/regulatory-information/search-fda-guidance-documents/evaluation-and-reporting-age-race-and-ethnicity-specific-data-medical-device-clinical-studies)   *Outlines FDA guidance for reporting age, race, and ethnicity data in clinical trials.*   - TransCelerate – [Diversity of Participants in Clinical Trials](http://www.transceleratebiopharmainc.com/initiatives/clinical-trial-diversification/)   *Provides tools and resources for improving diversity of clinical trial participants.*   - SCRS – [Diversity Site Assessment Tool](https://myscrs.org/dsat/)   *Evaluates capacity to accommodate needs of diverse trial participants via self-assessment tool.* |  |
| 1. Research team clearly communicates study risks and benefits to study participants in a manner that is accessible and culturally/linguistically appropriate. | - CTTI – [Recommendations for Informed Consent](https://ctti-clinicaltrials.org/wp-content/uploads/2021/06/CTTI_Informed_Consent_Recs.pdf)   *Provides recommendations for improving the informed consent process.*   - FDA – [Guidance for IRBs and Clinical Investigators: A Guide to Informed Consent - Information Sheet](https://www.fda.gov/RegulatoryInformation/Guidances/ucm126431.htm)   *Outlines FDA guidance on conducting and documenting informed consents.* | - Policies and procedures |
